# Supplementary material for: Soil Humus, Iron, Sulphate and Magnesium Content Affect Nectar Traits of Wild Garlic (Allium ursinum L.)
Source: Plants (Basel). 2021 Mar 22;10(3):597. doi: 10.3390/plants10030597 (PMC8004594; doi:10.3390/plants10030597)
Supplement: Supplementary file 1 [file plants-10-00597-s001.pdf]

SM Table 1. Different parameters of soil samples taken directly from around the roots of examined plants. The soil data used to make Figures 4-6.

**Site name**	**PA**	**pH (KCl)**	**pH (H_2O)**	**Humus (%)**	**$CaCO_3$ (m m^{-1} %)**	**Salt (m m^{-1} %)**	**Nitrate (mg kg^{-1})**
Ágfalva	57.3	6.4	6.6	5.1	0.10	0.05	56.8
sd	1.2	0.5	0.6	0.6	0.00	0.01	22.2
Bisse	50.7	6.2	6.6	4.1	0.10	0.01	23.9
sd	0.6	0.5	0.3	0.0	0.00	0.00	7.7
Bőszénfa	48.7	5.7	6.3	3.7	2.92	0.02	21.7
sd	2.5	1.3	1.0	0.6	4.88	0.02	6.1
Jánossomorja	52.0	7.3	7.5	4.5	8.27	0.08	43.6
sd	10.5	0.2	0.1	1.3	9.65	0.01	27.3
Lapis	48.7	4.9	5.6	4.4	0.10	0.01	18.2
sd	4.0	0.7	0.7	0.8	0.00	0.00	9.4
Lórév	18.4	2.3	2.4	1.9	1.66	0.01	11.2
sd	26.2	2.3	2.7	2.2	2.79	0.01	6.3
Pusztamarót	57.7	6.5	6.9	5.6	1.59	0.05	62.6
sd	4.0	0.7	0.5	0.3	2.58	0.02	27.1
Rajka	60.3	7.3	7.4	5.5	9.64	0.08	92.6
sd	1.5	0.1	0.1	0.7	1.07	0.01	34.0
Szenta	69.0	5.5	5.7	6.2	0.10	0.05	173.4
sd	10.4	0.6	0.6	0.5	0.00	0.04	140.9
Tatabánya	48.7	7.0	7.1	6.1	3.55	0.09	119.7
sd	3.1	0.2	0.1	0.3	5.61	0.01	52.4
Zalaistvánd	43.3	5.1	5.4	4.9	0.10	0.04	52.6
sd	4.5	0.2	0.2	0.7	0.00	0.03	14.8
Zalaszántó	54.7	7.1	7.2	3.8	1.61	0.03	50.8
sd	17.6	0.1	0.1	0.1	2.62	0.02	13.9
Zobákpuszta	75.0	7.0	7.2	5.6	3.09	0.07	52.2
sd	0.0	0.1	0.1	0.1	2.00	0.01	6.0

The first row next to each sampling site shows average values (n=3), and standard deviations (*sd*) are listed below.

SM Table 2. Mineral content of soil samples taken directly from around the roots of examined plants. The soil data used to make Figures 4-6.

Site name	Cu (mg kg^{-1})	Fe (mg kg^{-1})	K_2O (mg kg^{-1})	Mg (mg kg^{-1})	Mn (mg kg^{-1})	P_2O_5 (mg kg^{-1})	Zn (mg kg^{-1})	SO_4 (mg kg^{-1})
Ágfalva	0.25	672	362	453	620	165	2.5	130
sd	0.00	16	19	15	86	20	0.3	13
Bisse	0.25	864	308	416	695	140	0.1	65
sd	0.00	218	37	93	40	67	0.1	3
Bőszénfa	0.25	839	263	198	691	507	4.2	80
sd	0.00	248	119	28	307	553	7.2	2
Jánossomorja	0.25	279	1065	2154	288	117	0.8	219
sd	0.00	48	1043	663	99	47	1.2	207
Lapis	0.25	581	330	181	509	79	0.0	74
sd	0.00	23	152	12	142	17	0.0	14
Lórév	0.08	284	200	74	319	216	2.4	30
sd	0.14	281	114	94	184	293	4.2	39
Pusztamarót	0.25	456	475	857	436	59	2.8	133
sd	0.00	137	104	553	223	9	3.4	14
Rajka	0.25	367	463	4391	294	64	2.9	162
sd	0.00	24	87	290	9	19	2.0	19
Szenta	0.25	1971	220	597	206	261	5.7	267
sd	0.00	489	8	166	90	87	1.3	8
Tatabánya	0.25	503	371	261	366	233	4.6	180
sd	0.00	52	313	231	180	45	4.4	14
Zalaistvánd	0.25	533	136	297	286	74	0.02	61
sd	0.00	43	106	33	79	2	0.0	12
Zalaszántó	0.25	393	432	3025	261	126	0.0	51
sd	0.00	117	51	1215	65	57	0.0	5
Zobákpuszta	0.25	515	1176	347	328	161	6.0	67
sd	0.00	29	180	18	18	31	5.2	15

The first row next to each sampling site shows average values (n=3), and standard deviations (*sd*) are listed below.
